# Domoic Acid Oxidative Effects on the Microalgae *Phaeodactylum tricornutum*

**DOI:** 10.3390/life13030676

**Published:** 2023-03-02

**Authors:** Joaquin Cabrera, Susana Puntarulo, Paula Mariela González

**Affiliations:** 1 Facultad de Farmacia y Bioquímica, Universidad de Buenos Aires, Fisicoquímica, Buenos Aires CP 1113, Argentina; 2 Instituto de Bioquímica y Medicina Molecular (IBIMOL), CONICET-Universidad de Buenos Aires, Junín 956 (CP C1113AAD), Buenos Aires CP 1113, Argentina

**Keywords:** allelopathy, antioxidants, biotoxin, diatoms, harmful algae blooms, oxidative stress

## Abstract

Domoic acid (DA) is a natural occurring marine biotoxin. Oxidative stress generation due to DA exposure was reported in animals, but little is known on the phytoplankton community. The aim of this work was to verify whether exposure to DA in the marine diatom *Phaeodactylum tricornutum* favors reactive oxygen species (ROS) generation in the intracellular environment modifying its antioxidant capacity. Active species production, non-enzymatic antioxidant content, and antioxidant enzyme activities over the three growth phases of *P. tricornutum* exposed to 64 µM DA were evaluated. Results obtained in exponential growing cells showed a time-depending seven-fold increase in the 2′,7′ dichlorofluorescein diacetate dye oxidation rate. Superoxide dismutase and catalase activities showed a two-fold increase, and glutathione related enzymes activities were also significantly increased in treated diatoms as compared to controls. However, glutathione and ascorbate contents significantly decreased after incubation of the cells with DA. Similar effects were observed in latent and stationary phases of cell development. These results showed that DA could cause a severe oxidant-dependent impact on a non-toxic algae.

## 1. Introduction

Harmful algal blooms (HABs) threaten fresh water and marine ecosystems by the generation of hypoxic conditions and the production of toxins [1]. Climate change is affecting the environment and increasing the impact and frequency of HABs on aquatic ecosystems [2]. The increase in the natural environment of the content of nitrogen and phosphorus stimulates both the excessive growth of many types of algae and the prevalence of HABs [3]. These nutrients enter rivers and estuaries from many sources, such as improperly treated wastewater, fertilized lawns, and agricultural runoff, either from fertilized fields, animal wastes, or intense precipitation events [4]. It was described that long-term changes in water temperatures may allow some species of toxic algae to increase the seasonality of their blooms, or to expand to temperate zones [5]. The oceans function as a carbon sink, absorbing carbon dioxide (CO_2_) from the atmosphere. The continuous emission of CO_2_ has contributed to sea water acidification due to its behavior in solution [5]. The progressive acidification of the ocean could inhibit the growth of phytoplankton species that have calcium carbonate shells which are dissolved in acidic conditions, but could favor organisms without calcium carbonate shells, including harmful dinoflagellates and diatoms [5].

Domoic acid (DA) is a marine biotoxin produced by some diatom species, such as *Pseudo-nitzchia* sp., *Amphora coffeaeformis,* and *Nitzschianavis-varingica* [6,7]. In 2007, an important outbreak of this kind of HAB occurred, killing many species including seals, sea otters, dolphins, whales, and a large number of birds [8]. Exposure to the DA toxin leads to Amnesic Shellfish Poisoning (ASP). DA is an excitatory amino acid with a high affinity for propanoic acid receptors and kainate subclasses of glutamate receptors, which are present in the central nervous system and myocardium of vertebrates. In humans, DA causes the production of reactive oxygen species (ROS), neurological dysfunction, DNA damage, lipid peroxidation, energy depletion, mitochondrial damage, and cell death [3]. As a secondary effect of its neurotoxicity, the generation of oxidative stress due to DA exposure was reported in many animal species, such as bivalves, fishes, nematodes, and in aquatic and terrestrial mammals [9,10].

The overproduction of reactive species in photosynthetic and respiring cells is controlled by the presence of antioxidants, and by the radical scavenging biochemical reactions [11]. The antioxidant cellular defense network includes the activity of enzymes (e.g., catalase, CAT; superoxide dismutase, SOD; glutathione peroxidase, GPx; glutathione reductase, GR; glutathione S-transferase, GST), and hydrophilic (e.g., ascorbic acid, AH^−^; reduced glutathione, GSH), and lipophilic (e.g., α-Tocoferol, α-T; β-carotene, β-C) compounds [12]. Reports from Tian and Zhang [9] indicated that an antioxidant treatment suppressed the toxic effects of DA in the locomotion behavior of nematodes.

Positive (hormesis) and negative (oxidative stress) effects due to allelopathic interactions between plants and other photosynthetic organisms were registered [13]. Yang et al. [14] reported that in *Phaeodactylum tricornutum,* the allelochemical pesticide hydroquinone induced alterations in cell membrane permeability and mitochondrial membrane potential, and increased antioxidant enzyme activities, including SOD, CAT, GST, and GPx, followed by a reduction of the GSH content. Yang et al. [15], using the same diatom exposed to the allelochemical pesticide ethyl 2-methyl acetoacetate, showed oxidative damage induction and changes in antioxidant activities. However, little is known about the possible effects that other microalgae may have on non-toxic microalgae species. Some species of *Alexandrium sp*. were reported to generate allelopathic effects on microalgae. Inhibitory effects of *A. pacificum* filtrate on *Talassiosira pseudonana* was observed by Mao et al. [16]. Co-culturing influenced the expression of *T. pseudonana* genes related to photosynthesis, oxidative phosphorylation, antioxidant system, nutrient absorption, energy metabolism, alterations in the photosystem II, increased SOD activity, and malonyldialdehyde content [16]. Allelopathic effects were also observed with *Pseudo-nitzschia* co-cultured with dinoflagellates, raphidophytes, haptophytes, and cryptophytes [17,18]. Xu et al. [18] reported that a filtered fraction from a *P. multiseries* culture reduced cell density of the dinoflagellate *Akashiwo sanguinea*. A possible allelochemical effect of DA was proposed by Bates et al. [19]. Van Meerssche and Pinckney [20], using microcosm experiments conducted in a high salinity and nutrient depleted ecosystem, showed that an increase in salinity lead to increased inhibition of the growth rate of diatoms and cryptophytes exposed to dissolve DA (dDA). Under low salinity conditions, similar results on a natural phytoplankton community were reported by Van Meerssche et al. [21].

*P. tricornutum* is a pennate diatom frequently used in aquaculture due to its culturing simplicity, lack of toxicity, high nutritional value, and its digestible cell wall that makes nutrients available [22]. This non-toxic diatom, which possesses three morphological forms (bilateral, oval, and tri-radiated), is widely distributed and cohabits in environments with *Pseudo-nitzschia* sp. In this scenario, the toxin produced by *Pseudo-nitzschia* sp. could be released in the water and reach other organisms. The input of DA into cells could generate ROS, which could then affect the welfare of other aquatic life in the environment. The hypothesis of this work was that the exposure of *P. tricornutum* to DA triggers the generation of reactive species in the intracellular environment, modifying its cellular redox balance and antioxidant capacity. Under laboratory conditions, the generation of reactive species, assessed as the oxidation rate of the dye 2′,7′ dichlorofluorescein diacetate (DCFH-DA), the GSH and AH^−^ content, and the activity of antioxidant enzymes (SOD, CAT, GPx, GR, and GST) were evaluated during the growth of *P. tricornutum* cultures exposed to DA.

## 2. Materials and Methods

### 2.1. Biological Material

The study was conducted with a microalgae strain of *P. tricornutum* from an axenic collection culture of the Aquaculture Laboratory from the Marine and Costal Research Institute (IIMyC), Mar del Plata, Argentina. The algae were cultured in an F/2 Guillard medium [23] in filtered (0.25 µm) sea water of 33 psu. Semi-continuous cultures were kept in 250 mL glass containers. The cultures were exposed to 65.4 W/m^2^ photosynthetically active radiation (PAR) with fluorescent tubes of 40 W at 18 °C in a culture chamber Precision PS Scientific Co.

### 2.2. Growth Phases Characterization

An inoculum with a cell density of 2.5 × 10^6^ cells/mL of *P. tricornutum* in 250 mL of F/2 medium was grown for 15 days and changes in the number of cells, optical density (OD), and the content of total chlorophyll (T Chl) were determined. The cell counting was performed in a Neubauer chamber under a light microscope Numak with a 400× magnification. The total number of cells/mL was calculated according to FAO [24]. The OD was measured spectrophotometrically in a spectrophotometer (Beckman DU 7400 U*V*/*V*is) at λ = 600 nm and T Chl was measured at different λ (630, 647, 664, and 691 nm) [25,26]. The T Chl content in the methanol extracts was calculated according to Ritchie et al. [26] following the equation: Total Chl = 21.3877 A_630_ + 10.3739 A_647_ + 5.3805 A_664_ + 5.5309 A_691_ (±0.0056). This procedure was employed to determine the three growth phases of *P. tricornutum* cultures in control cells.

### 2.3. Microalgae Exposure to DA

Cells of *P. tricornutum* were concentrated by centrifugation at 9300× *g* for 10 min at 4 °C, and were supplemented with DA (Sigma D6152, purity ≥ 90% HPLC quality) to a final concentration of 64 µM. This DA concentration was selected according to the regulations on the values of toxin content in non-toxic sea food for humans in most countries [27]. All determinations were performed in latent (LAG, 4 days of growth), exponential (EXP, 7 days of growth), and stationary (STA, 12 days of growth) phases. After DA exposure, the cells were washed with F/2 medium and were centrifuged three times at 9300× *g* for 10 min at 4 °C. Pellets with cells were preserved at −80 °C until processing. The cells were incubated during 2, 5, 7, and 12 min, either in the presence of 64 µM DA or in its absence (controls).

### 2.4. Diatom Electron Microscopy

Cells collected after centrifugation with the refrigerated centrifuge Eppendorf 5415R at 9000× *g* during 10 min were fixated in 2% (*v*/*v*) glutaraldehyde for 2 h at 4 °C. Samples were then fixated with 1% (*w*/*v*) osmium tetroxide (pH 7.2) for 1 h, and dehydrated with 50, 70, 80, 90, and 95% (*v*/*v*) ethanol and propylene oxide for 10 min. Thin sections were cut with glass or diamond knives on a microtome, stained with uranyl acetate, and examined with a JEM 1200 EX II electron microscope (Joel Ltd., Tokyo, Japan) of the Central Service of Electron Microscopy of the Faculty of Veterinary Sciences from National University of La Plata, Argentina. Average cell size in control and treated cultures was determined by the analysis of transmission electron microscopy (TEM) images using the ImageJ software [28].

### 2.5. Determination of Cellular DA Content

The DA content was measured by HPLC (Waters and Perkin-Elmer ISS200) with a UV/Electrochemical detector following the protocol of Lawrence et al. [29] with modifications by Quilliam et al. [30]. The used DA standard was of 90% (*v*/*v*). A C-18 SUPELCO stainless steel chromatographic column (15 cm × 4.6 mm internal diameter and a 5 µm particle diameter) was used for reverse phase chromatography. The operating conditions were isocratic system, pH 2.5, phosphoric acid:acetonitrile (93:7) mobile phase, 1 mL/min flow, 20 µL injection volume, UV detection (λ = 242 nm) and 10 min runtime.

### 2.6. DCFH-DA Oxidation Rate

The oxidation rate of DCFH-DA was measured with a fluorometric assay following the oxidation of the DCFH-DA dye in a fluorometer (F-3010 Hitachi) at λ_ex_ = 488 nm and λ_em_ = 525 nm [31]. The F/2 medium containing the diatoms was centrifuged and washed. The supernatant was removed, and the pellet was sonicated using a Branson Sonifier 450 with a frequency of 30% duty cycle during 30 s on ice, between 3 or 4 input pulse and was incubated with 10 µM DCFH-DA during 45 min at 18 °C. After this incubation, the samples were centrifuged, and the DCFH-DA oxidation rate was measured following the protocol described by Hernando et al. [32].

The identity of the reactive species that contributed to the oxidation of the dye was studied using reactive species quenchers. Scavengers were added after the pellet sonication, and the samples were incubated for 45 min at 18 °C. Then, the homogenate was centrifuged and the fluorometric measurement was performed in a microplate reader (Varioskan Lux Thermo Fisher Scientific, Waltham, Massachusetts, USA). The addition of the enzymes SOD (300 U/mL) and CAT (500 U/mL), dimethyl sulfoxide (50 mM, DMSO), the antioxidant GSH (5 mM), and the iron chelator deferoxamine (50 µM, DF) were tested in the presence and absence of DA. Three replicates of each algal culture were tested. The percentage of inhibition of the oxidation rate of the dye by the addition of these scavengers was calculated as the decrease in the oxidation rate of DCFH-DA (∆ Oxidation rate) after incubation with each agent.

### 2.7. Determination of the Antioxidant Enzyme’s Activities

Cells were homogenized in a 40 mM potassium phosphate buffer with 120 mM KCl (pH 7.4) and were centrifuged at 9000× *g* for 10 min to obtain the supernatant. CAT activity was assayed spectrophotometrically in Beckman DU 7400 U*V*/*V*is (λ = 240 nm) by the decomposition of hydrogen peroxide (H_2_O_2_) in a reaction mixture consisting of 50 mM potassium phosphate buffer (pH 7.0) containing 10 mM H_2_O_2_ [33]. CAT activity was expressed as units per million cells (U/10^6^ cells; U = mM/min). SOD activity was assayed spectrophotometrically by the cytochrome *c* detection system, where superoxide anion (O_2_^−^) is enzymatically generated by the xanthine-xanthine oxidase system, and it reduces the cytochrome *c* yielding a product which absorbs at λ = 550 nm [34]. The reaction mixture consisted of 50 mM potassium phosphate buffer with 0.1 mM EDTA (pH 7.8), 500 µM xanthine prepared in 1 mM NaOH, and 5 µM xanthine oxidase to give approximately 0.025 absorbance units of increase/min. The amount of SOD able to inhibit the cytochrome c reduction rate by 50% was defined as one unit of the enzyme [35]. The activities of GST, GPx, and GR were determined following spectrophotometric methods (λ = 340 nm) described by Paglia and Valentine [36], Habig et al. [37], and Carlberg and Mannervick [38], respectively, in a microplate reader. All three enzymatic activities were expressed as U/10^6^ cells (U = mM/min).

### 2.8. Determination of AH^−^ Content

AH^−^ content was measured by reverse phase HPLC with electrochemical detection. The samples were homogenized in 10% (*w*/*v*) metaphosphoric acid (MPA) according to Kutnink et al. [39]. The column used (Supelcosil LC-8; 15 cm × 4.6 mm, particle size 3 μm) was stabilized with a 0.8% MPA mobile phase, and the chromatography was carried out with a continuous flow of 1.2 mL/min. Standard AH^−^ (Sigma A-7506, 98% purity) was used to quantify the antioxidant content.

### 2.9. Determination of GSH Content

GSH content was determined by reverse phase HPLC with electrochemical detection [40]. A Supelcosil LC-18-DB column (4.6 × 250 mm, particle size 5 μm) was used. The column was stabilized with a 20 mM NaH_2_PO_4_ pH 2.7 mobile phase, and the detector was an electrochemical ESA Coulochem II with ESA model 5011 analytical cell. The samples were measured with +0.45 V and 0.80 V applied potential and 1.2 mL/min flow. Samples were homogenized in HClO_4_ (20,000 cell/mL) with 2 mM EDTA. Afterwards, they were centrifuged at 9300× *g* for 20 min at 4 °C, and then were injected into the HPLC after filtration through 0.22 µm nylon membranes. The antioxidant content was measured using a calibration curve with standard GSH (Sigma G-4251, 98–100% purity).

### 2.10. Statistical Analyses

Data were expressed as mean ± S.E.M. of three independent cultures, with duplicates for each condition. The used of one-way ANOVA of one-tailed with no adjustments for multiple comparisons was done for the data analyses comparing differences in each growth phase and between them. Statistical tests were carried out using Statview 5.0 for Windows, ANOVA, SAS Institute Inc.

## 3. Results

### 3.1. Growth Phases and DA Exposure Characterization

**Figure 1:**
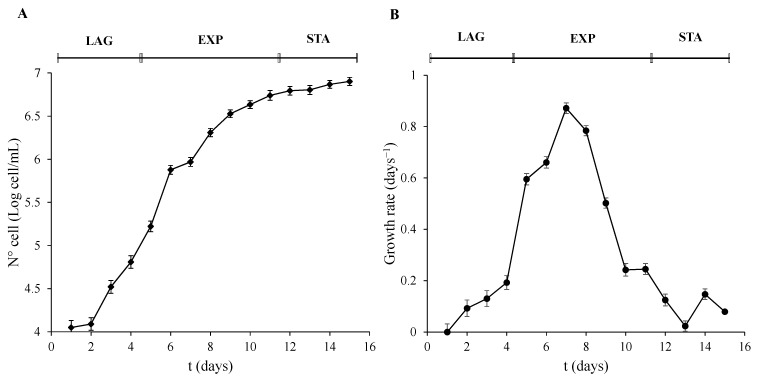
Growth phases characterization of *P. tricornutum*. (**A**) Cell number expressed as Log of cell/mL, and (**B**) growth rate was calculated as µ = (LnN − LnN_0_)/(t − t_0_) were N means N° cell/mL at a determined t time, and N_0_ means N° cell/mL at an initial t_0_ time. Error bars indicate standard error of the mean of three independent cultures, with duplicates for each condition.

Three different stages of the culture were observed. The LAG phase lasted the first 4 days. The parameters of cell number, OD, and T Chl were not significantly (*p* > 0.01) altered during this period. However, they were significantly (*p* < 0.001) increased after 5 days of development until day 11. This phase was recognized as the EXP phase of growth. Finally, 12 days after inoculation, the STA phase of the algal culture growth was reached and was significantly different to the EXP phase (*p* < 0.001). Even though the T Chl and OD did not show differences in the growth trend compared to the EXP phase, the transformation of the data of the cell number (Figure 1A) and the decrease in the growth rate (Figure 1B) was used to determine the STA phase. Cells were collected on days 4, 7, and 12, and were taken as representative of the three phases, LAG, EXP, and STA, respectively.

**Figure 2:**
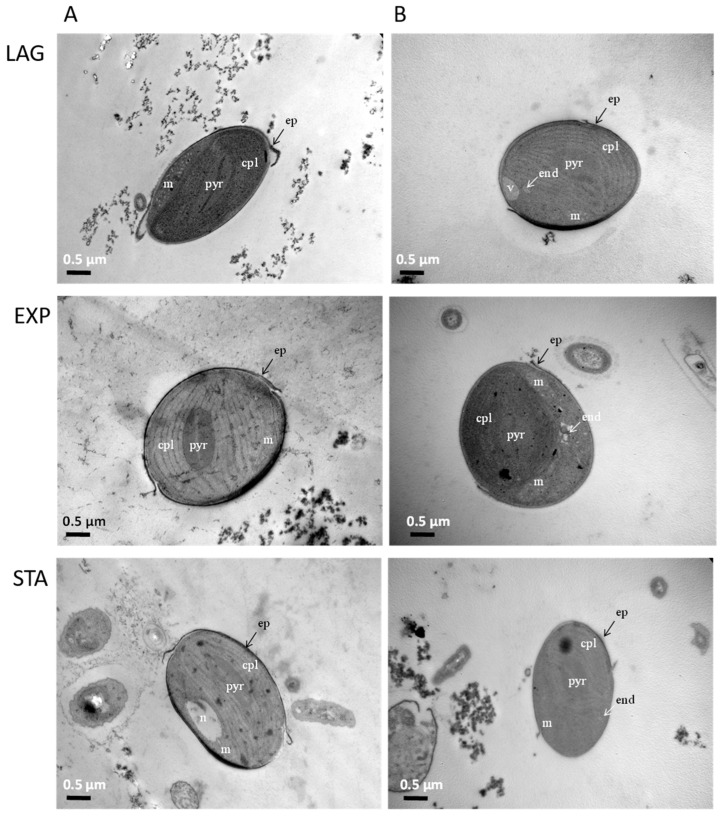
Subcellular structures of *P. tricornutum* during the three growth phases: **Left panels A:** control algae, and **Right panels B:** cells exposed to DA for 12 min. n: nucleus, m: mitochondria, cpl: chloroplast, pyr: intraplastidial pyrenoid, v: vacuole, end: endosome, ep: epivalve.

The bilateral form of *P. tricornutum* was the predominant morphology found in the algal culture. This shape has the particularity of showing a single shell of silica (Figure 2) which can be observed in the cross sections of the cells in the three growth phases. The internal morphology in these cells showed a single chloroplast (cpl) occupying the largest percentage of the cell with an intraplastidial pyrenoid (pyr). Other cellular structures such as mitochondria (m), nucleus (n), and vacuole (v) were also seen in the TEM pictures. Either control cells (Figure 2A) or algae exposed to DA (Figure 2B) had shown the same structures. However, in DA exposed cells an endosome-like (end) structure appeared in all three growth phases.

**Figure 3:**
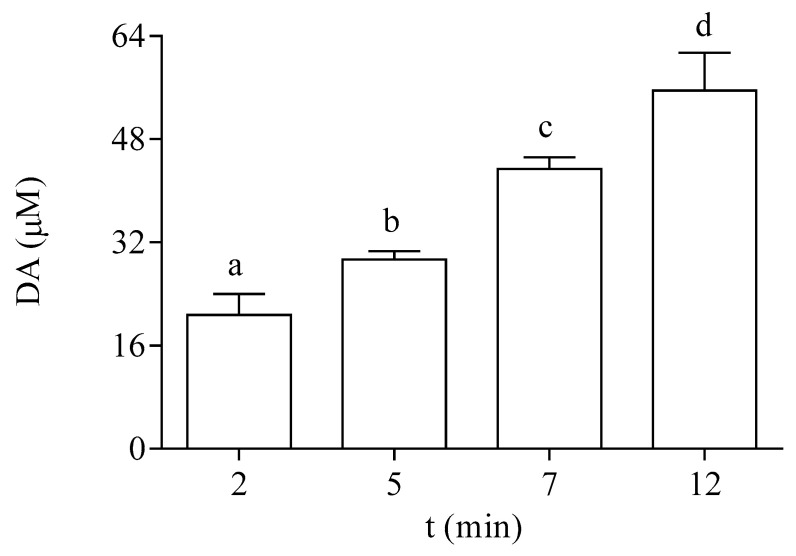
Intracellular DA concentration in EXP cells of *P. tricornutum* exposed at different times to 64 µM DA. Lowercase letters show significant differences between different exposure times. ANOVA (*p* < 0.001). Error bars indicate standard error of the mean of three independent cultures, with duplicates for each condition.

*P. tricornutum* cells actively incorporated DA from the medium. Data shown in Figure 3 indicate that the exposure of the microalgae to 64 µM DA for 2, 5, 7, and 12 min resulted in a significant increase in the intracellular content of the toxin at the EXP phase. After 12 min, the cellular concentration of DA represented 87% of the DA concentration added in the external medium.

### 3.2. Screening of Active Species Generation in EXP Phase Cells Exposed to DA

**Figure 4:**
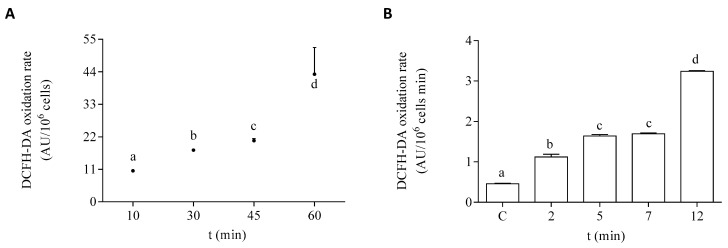
DCFH-DA oxidation rate by *P. tricornutum* cells in the EXP growth phase: (**A**) oxidation rate of the dye by control cells measured at different times of incubation in the presence of DCFH-DA, and (**B**) oxidation rate of the dye by cells incubated for 45 min with DCFH-DA after the treatment with 64 µM DA. Lowercase letters show significant differences between different exposure times. ANOVA (*p* < 0.001). Error bars indicate standard error of the mean of three independent cultures, with duplicates for each condition.

The oxidation rate of DCFH-DA in the EXP phase of growth of the microalgae is shown under control conditions and after exposure to DA in Figure 4. To select the optimal time of exposure to the dye, control cells in the EXP phase were incubated with it at different time points. The selected incubation time used for the experimental protocols employed in this study was 45 min (Figure 4A). The effect of DA was studied at different times of the toxin exposure in EXP phase cells. After 12 min of exposure to the toxin, the dye oxidation rate was increased by seven-fold, as compared to cells incubated in the absence of DA (Figure 4B). In both controls and DA incubated cells, the addition of scavengers of active species to the algae homogenates significantly decreased the oxidation rate of DCFH-DA, as compared to the basal levels (Table 1). The oxidation rate of DCFH-DA by the exposure of the cells to DA was sensitive to the presence of all the tested scavengers under the experimental conditions employed here. Higher percentages of inhibition of the oxidation rate of the dye in the algae exposed to DA were observed with the addition of CAT, DF, and GSH.

### 3.3. Redox Response of Cells Exposed to DA during the EXP Phase of Growth

**Figure 5:**
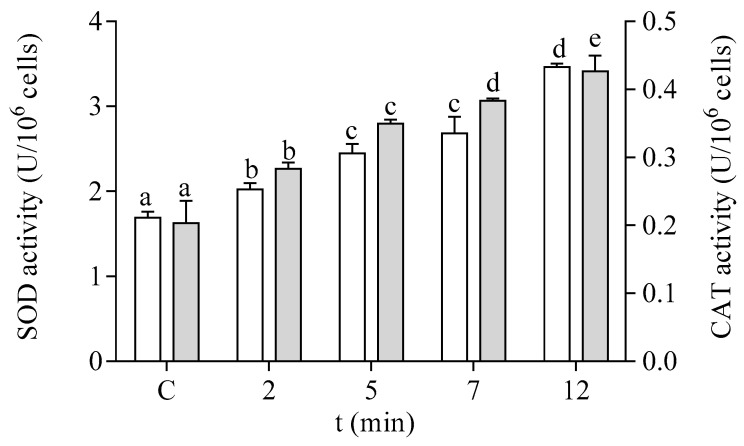
Effects of the exposure to 64 µM DA on the SOD (□) and CAT (■) activities of cells of *P. tricornutum* in the EXP phase of growth. Lowercase letters show significant differences between different times of exposure to DA. ANOVA (*p* < 0.001). Error bars indicate standard error of the mean of three independent cultures, with duplicates for each condition.

**Figure 6:**
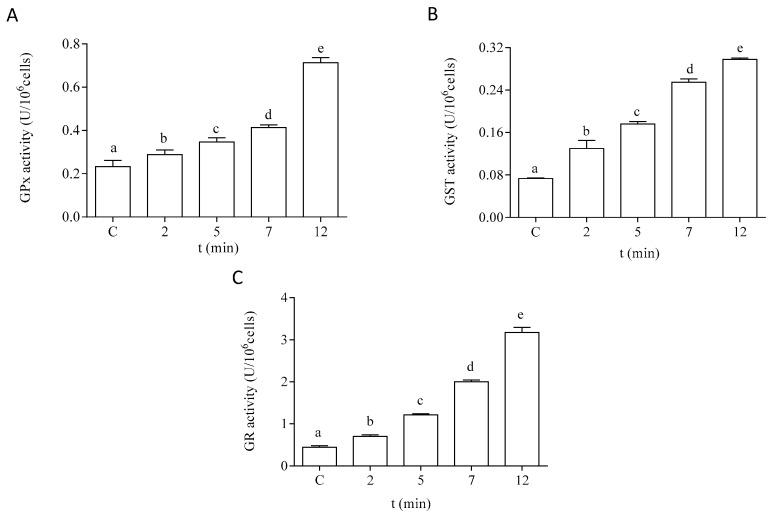
Effects of the treatment with 64 µM DA on the activities of the GSH metabolism-related enzymes on *P. tricornutum* cells in the EXP phase of growth: (**A**) activity of GPx, (**B**) activity of GST, and (**C**) activity of GR. Lowercase letters show significant differences between different exposure times. ANOVA (*p* < 0.001). Error bars indicate standard error of the mean of three independent cultures, with duplicates for each condition.

The antioxidant capacity of the cells of *P. tricornutum* was modified after in vitro exposure to DA. The effect of DA on the activity of the antioxidant enzymes SOD and CAT is shown in Figure 5. Both enzymatic activities were increased by two-fold after 12 min of exposure to 64 µM DA in the microalgae cells at the EXP phase of development. The enzymes related to GSH metabolism also showed a significant increase in their activities in a time-dependent manner after treatment with DA (Figure 6). The activities of GPx and GST were increased by three-fold under these experimental conditions, as compared to control cells incubated in the absence of the biotoxin (Figure 6A,B, respectively). GR activity was increased by seven-fold after 12 min of incubation with DA as compared to controls (Figure 6C).

**Figure 7:**
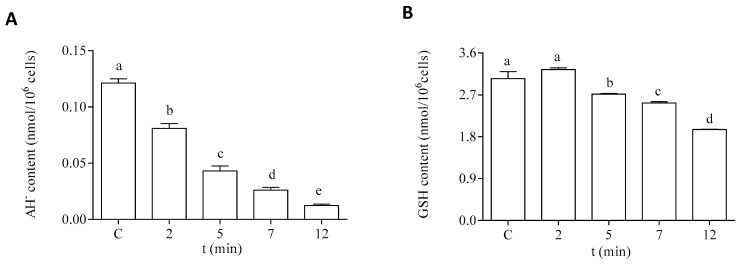
Intracellular content of hydrophilic antioxidants in the EXP phase cells of *P. tricornutum* after being exposed to 64 µM DA during different times. (**A**) Content of AH−, and (**B**) content of GSH. Lowercase letters show significant differences between the different exposure times. ANOVA (*p* < 0.001). Error bars indicate standard error of the mean of three independent cultures, with duplicates for each condition.

Regarding the hydrophilic antioxidants, both AH^−^ and GSH content were significantly decreased in the cells after exposure to DA (Figure 7). The intracellular content of AH^−^ showed a significant decrease after 2 min of incubation with DA (Figure 7A). An 80% reduction of this antioxidant was observed after 12 min of exposure to the biotoxin, as compared to the control levels. Moreover, a significant reduction (30%) in the intracellular GSH content was observed after 12 min of exposure to DA, as compared to cells incubated under control conditions (Figure 7B).

### 3.4. Cellular Redox Responses after Treatment with DA by Cells in LAG and STA Growth Phases

Cells of *P. tricornutum* in LAG and STA growth phases showed similar effects in their oxidative parameters to those described for cells in the EXP phase (Table 2). *P. tricornutum* cells in LAG, EXP, and STA growth phases showed between 84% and 89% of DA incorporation at 12 min of DA exposure. However, the active species generation was significantly lower in the LAG and STA growth phases, as compared to the generation in cells in the EXP phase of growth. In cells of LAG and STA growth phases, the DCFH-DA oxidation rate was significantly higher after the DA treatments, as compared to control conditions (two-fold). Additionally, significant increases in the antioxidant enzyme activities were registered by the exposure to DA. Both SOD and CAT activities showed a three-fold increase in DA-treated algae (Table 2). The enzymes related to GSH metabolism (GPx, GR, and GST) also increased their activities in algae exposed to DA, as compared to controls by five-, seven-, and four-fold, respectively, in the LAG phase (Table 2). The same tendency was observed in the STA phase, except for the activity of the GPx enzyme which increased by 11-fold, as compared to controls (Table 2). CAT and GPx activities were found to be more significantly increased in DA-exposed cells in LAG and STA phases than in cells in the EXP growth phase. The cells exposed to DA showed significant decreases of the content of hydrophilic antioxidants (GSH and AH^−^) in LAG and STA phases, as compared to non-treated diatoms (Table 2). Cells in the LAG phase showed higher decreases in both parameters, especially in the content of AH^−^ (18-fold lower than control cells) (Table 2).

## 4. Discussion

It is believed that the increase in the content of the biotoxin DA is closely related to the oscillation (spring and summer) of the warm phases of the ocean, and events such as El Niño. Moreover, according to McKibben et al. [41], the northern coast of California showed a climatic regulation of DA in mussels in the last 20 years.

In the present work, DA was studied as a potential factor for oxidative alterations in *P. tricornutum,* given its possible allelochemical role [18]. Endosome-like vacuoles observed in *P. tricornutum* DA-exposed cells could be the result of a possible allelochemical function of the biotoxin. Similar vacuoles were reported by Zheng et al. [42] during the interaction of the same diatoms and the potentially toxic dinoflagellate *Alexandrium tamaerense*. The co-culturing experiment generated an inhibition in *P. tricornutum* growth, and microscopic evidence indicated the presence of chloroplastic and mitochondrial damage. Energy metabolism was also shown by down regulation of RNAseq genes involved in glycolysis, tricarboxylic acid cycle, β-oxidation, carbon fixation, and oxidation phosphorylation. In contrast, genes involved in endocytosis and in the transporter ABCB1 were upregulated. Moreover, in TEM micrographs the presence of an endosome was observed, suggesting that *P. tricornutum* could be sensitive to the release of certain allelochemicals from *A. tamarense*.

Previous studies [43] have shown that when the microalgae *P. tricornutum* were incubated for 12 min with DA 64 µM and buffer washed, the exposure to DCFH-DA for 60 min resulted in a significantly higher oxidation rate of the dye in the extracellular medium, as compared to control cells. Cellular cultures exposed to DA in the EXP phase of development showed a three-fold increase in the DCFH-DA extracellular oxidation rate, as compared to control cells. Further analysis on the nature of the involved species, studied employing quenchers, indicated that ROS content in the extracellular medium was decreased by 31% in the presence of SOD, 59% by CAT, 44% by DMSO, 22% by DF, and 98% by the addition of GSH. Similar effects were observed with cells in the three phases of growth. These results suggested that the exposure of *P. tricornutum* to DA leads to an increase in the production of ROS that can be released to the external medium. However, the half-life of this ROS is very short, which makes it difficult to reach other organisms and be responsible for substantial damage. Thus, ROS by themselves did not seem to be a critical contribution as compared to DA by itself, since cellular membranes are permeable to the toxin, as shown in Figure 3.

Furthermore, Bai et al. [13] suggested that oxidative stress could participate in the allelopathic response due to overproduction of ROS and alterations in the cellular antioxidant system. In this regard, the results reported here showed that the DA induced redox responses in *P. triconutum* in the three growth phases of the microalgae. The DCFH-DA oxidation rate, a general indicator of the cellular oxidative metabolism [44], was increased in DA exposed cells in a time-dependent manner. This fact can be directly related to the increase in the intracellular concentration of DA depending on the incubation time. Even more, the inhibitory effect on the DCFH-DA oxidation rate by the scavengers allowed us to infer the identity of the reactive species, implicated in the oxidation of the dye, that were triggered by the biotoxin [45]. The comparison among the effects of the tested oxidative radical scavengers showed that the addition of CAT, GSH, and DF produced the highest percentage of inhibition of the DCFH-DA oxidation rate in the diatoms exposed to DA. GSH is a non-specific antioxidant at the hydrophilic cellular level, and CAT and DF are involved in the metabolism of H_2_O_2_ and iron (Fe), respectively [4]. Thus, an increase in H_2_O_2_ generation and alterations in the Fe oxidative metabolism could be suggested as the main effects of the exposure of the microalgae to the biotoxin [46]. The addition to the experimental assay of the SOD enzyme also significantly decreased the oxidation rate of DCFH-DA in DA exposed cells. Since this enzyme is involved in the dismutation of O_2_^−^ [47], it could be suggested that an increase in the generation of this radical could also be relevant to the DA oxidative effects reported here.

Other antioxidant enzyme activities (CAT, GPx, and GR) are affected by the oxidative stress produced by exposure to DA [48]. The activities of all the antioxidant enzymes tested showed increases with DA exposure in a time-dependent manner. The elevation of antioxidant enzyme activities indicates the triggering of detoxification processes to face the enhanced formation of reactive species in plant cells [49]. Furthermore, hydrophilic antioxidant compounds, such as GSH and AH^−^, also seem to be involved in this redox response as protective agents against the produced oxidants. Redox responses to oxidative stress induced by biotoxins have been reported in the microalgae *Scenedesmus abundans* by Kaur et al. [50]. Oxidative stress was induced in *S. abundans* monocultures by the exposure of cell-free filtrate of the bloom-forming cyanobacteria *Mycrocistys aeruginosa.* A growth reduction of the green algae was shown during the initial stage of the experiment, but after 7 days, oxidative stress in *S. abundans* was reduced by the induction of both enzymatic and non-enzymatic antioxidants, such as SOD, CAT, GR, ascorbate peroxidase, AH^−^, and GSH.

Although cells of *P. tricornutum* in the three growth phases showed similar responses to DA exposure during 12 min, some parameters were significantly different between them. The major increase in the DCFH-DA oxidation rate after the exposure to the biotoxin was observed in the EXP growth phase. The antioxidant defense also exhibited significant differences. The activity of the antioxidant enzymes CAT and GPx were significantly increased in the LAG and STA phases, as compared to DA treated cells in the EXP growth phase. Besides that, microalgae in the EXP and STA growth phases showed major increases in GR activity after DA exposure. Thus, the higher decrease in GSH content observed in the LAG phase, as compared to the EXP phase of development, could reflect the significant enhancement in this enzymatic activity. Moreover, cells exposed to DA in the LAG phase of growth also exhibited a higher decrease in AH^−^ content, as compared to cells in the EXP growth phase. The different behavior observed among the growth phases could be influenced by the diverse nutrient availability and metabolism in each phase, being more active in the EXP phase [51,52]. To assess the magnitude of the ability of the antioxidant defense network to control the oxidant effects of DA exposure in the cells in the three phases of development, damage markers should be evaluated in the future.

Regarding the cellular Fe oxidative metabolism, Rue and Bruland [53] suggested that toxigenic effects of *Pseudo-nitzschia* species might be related to the role of DA as a trace metal chelator. DA showed the ability to capture Fe at low concentrations in the presence of Cu in the medium [17,54]. DA could bind with particulate Fe(III) in the water, and by its oxidation release soluble Fe(II), therefore being bioavailable for *Pseudo-nitzschia* [3,55]. The formation of a Fe-DA complex could play an active role in the increase of ROS generation, both in producing and non-phycotoxin producing species [56]. Studies with dDA and exudates from toxic *Pseudo-nitzschia* sp. suggested a possible allelopathic effect of DA in Fe-enrichment conditions in laboratory cultures [17,18]. Even more, Prince et al. [54] identified growth inhibition of the diatom *Skeletonema marinoi* induced by the direct addition of dDA in an Fe depleted medium. Furthermore, it should be considered that this Fe exposure could trigger a form of regulated cell death (RCD) known as ferroptosis, that is initiated by oxidative changes in the microenvironment that is under regulation by glutathione peroxidase 4 (GPx4), and inhibited by lipophilic antioxidants and Fe chelators [57]. This form of RCD is driven by the toxic accumulation of lipid hydroperoxides, and has a necrotic morphology [58] and the loss of activity of the lipid repair enzyme GPx4, which is followed by an accumulation of lipid-based ROS [14]. Future studies should be conducted in this regard.

## 5. Conclusions

**Figure 8:**
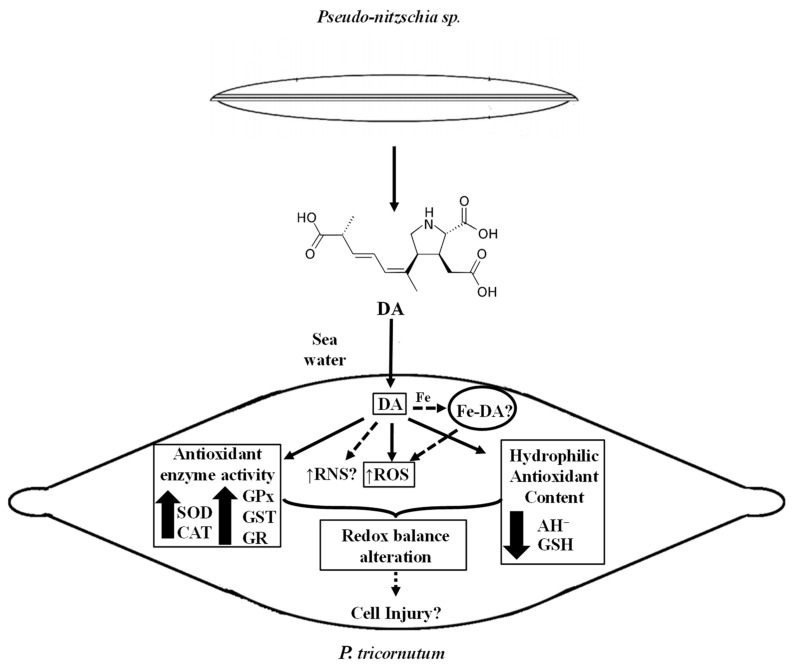
Schematic diagram showing a conceptual model according to de data presented in this study. Measured parameters affected by the DA exposure of *P. tricornutum* cells are indicated with a rectangle. Non-continuous lines refer to possible other oxidative effects of DA in *P. tricornutum* cells in their natural environment.

The evidence presented here reveals the oxidative effects caused by DA exposure to a non-toxic algae. The schematic diagram shown in Figure 8 is a conceptual model according to the results obtained in this study. An oxidative scenario triggered by the biotoxin is proposed here. DA is released into the environment and could be further incorporated into the diatom. Then, it could lead to the generation of active species, such as ROS, mainly O_2_^−^ and H_2_O_2_, according to the scavenging experiments. However, other reactive species not tested here, such as reactive nitrogen species (RNS), could also be involved in the alteration of the oxidative balance in the *P. tricornutum* cells. These aspects need further investigation.

The experimental data shown here also suggested that the increase in reactive species generation activates the antioxidant defense network, improving the antioxidant enzyme activities (SOD, CAT, GPx, GST, and GR), and reducing the content of hydrophilic antioxidants (AH^−^, GSH). Moreover, part of the cellular defense mechanism could include compartmentalization of the biotoxin. However, DNA, protein, and lipid damage could still be produced by exposure of the cell to DA, since the response of the antioxidant defense is not fully efficient to prevent cellular injury. Moreover, the possible catalytic effect of the complex Fe-DA, as part of the labile Fe pool, should be explored since it could induce redox responses in the cells. Although there is still much to investigate about the biotoxin role in non-target photosynthetic organisms, this work is the first report on the redox effects generated in organisms of the phytoplankton community by DA produced during HABs.

## Figures and Tables

**Table 1:** Effect of active species scavengers on the DCFH-DA oxidation rate by *P. tricornutum* homogenates either in the presence (+DA) or in the absence (−DA) of DA in the cells during the EXP growth phase.

DCFH-DA Oxidation Rate (AU/10^6^ Cells min)										
	−DA			+DA			Δ Oxidation Rate			Inhibition
Control	9.5	±	0.3	15.7	±	0.5 ^b^	6.6	±	0.2	
SOD	8.5	±	0.5 ^a^	13.0	±	0.3 ^a,b^	4.6	±	0.3 ^a^	30%
CAT	8.91	±	0.04 ^a^	10.3	±	0.1 ^a,b^	1.4	±	0.1 ^a^	78%
DMSO	9.1	±	0.2	13.8	±	0.7 ^a,b^	4.7	±	0.5 ^a^	29%
GSH	6.5	±	0.2 ^a^	8.2	±	0.4 ^a,b^	1.7	±	0.2 ^a^	74%
DF	8.6	±	0.2 ^a^	11.1	±	0.3 ^a,b^	2.5	±	0.4 ^a^	62%

^a^ Significantly different from the DCFH-DA oxidation rate by the cellular homogenate in the absence of scavengers; ANOVA, (*p* < 0.0001). ^b^ Significantly different from the DCFH-DA oxidation rate by the cellular homogenate in the absence of DA; ANOVA, (*p* < 0.0001). DCFH-DA stands for 2′,7′ dichlorofluorescein diacetate; DA, for domoic acid; SOD, for superoxide dismutase; CAT, for catalase; DMSO, for dimethyl sulfoxide; GSH, for reduced glutathione; DF, for deferoxamine.

**Table 2:** Oxidative parameters tested on *P. tricornutum* cells in LAG and STA phases of growth either under control (−DA) or in cells incubated during 12 min with 64 µM DA (+DA).

	LAG						STA					
	−DA			+DA			−DA			+DA		
% DA uptake	----			85			----			89		
DCFH-DA oxidation rate (AU/10^6^ cells min)	0.67	±	0.01 ^b^	1.41	±	0.03 ^a,b^	0.87	±	0.02 ^c^	2.08	±	0.01 ^a,c^
SOD activity (U/10^6^ cells)	2.2	±	0.4 ^c^	3.5	±	0.1 ^a^	1.6	±	0.1 ^d^	3.5	±	0.2 ^a^
CAT activity (U/10^6^ cells)	0.24	±	0.04	0.60	±	0.04 ^a,c^	0.23	±	0.04	0.670	±	0.05 ^a,c^
GPx activity (U/10^6^ cells)	0.20	±	0.01	1.03	±	0.09 ^a,c^	0.19	±	0.05	1.02	±	0.06 ^a,c^
GST activity (U/10^6^ cells)	0.06	±	0.01	0.23	±	0.01 ^a,b^	0.058	±	0.008	0.251	±	0.004 ^a,c,d^
GR activity (U/10^6^ cells)	0.27	±	0.03 ^c^	1.98	±	0.15 ^a,c^	0.26	±	0.02 ^c^	3.1	±	0.2 ^a,d^
AH^−^ content (nmol/10^6^ cells)	0.108	±	0.004 ^c^	0.006	±	0.0003 ^a,c^	0.19	±	0.02 ^b,d^	0.024	±	0.003 ^a,c,d^
GSH content (nmol/10^6^ cells)	1.74	±	0.08 ^b^	1.13	±	0.3 ^a,b^	2.36	±	0.09 ^c,d^	1.92	±	0.01 ^a,d^

% DA uptake: percentage of intracellular DA as compared to the toxin content in the incubation medium. ^a^ Significantly different from the −DA condition; ANOVA, (*p* < 0.001). ^b^ Significantly different from the cells in the EXP growth phase at the same condition; ANOVA, (*p* < 0.001). ^c^ Significantly different from the cells in the EXP growth phase at the same condition; ANOVA, (*p* < 0.01). ^d^ Significantly different from the cells in the LAG phase at the same condition; ANOVA, (*p* < 0.01). DA stands for domoic acid; DCFH-DA, for 2′,7′ dichlorofluorescein diacetate; SOD, for superoxide dismutase activity; CAT, for catalase activity, GPx, for glutathione peroxidase activity; GST, for glutathione-S-transferase activity; GR, for glutathione reductase activity; AH−, for ascorbate content; GSH, for reduced glutathione content.

## Data Availability

The data presented in this study are available on request to the corresponding author.
